# Lumbar Tuberculous Spondylodiscitis with Abscess Formation

**DOI:** 10.1590/0037-8682-0502-2023

**Published:** 2024-02-05

**Authors:** Handan Alay, Bahar Yılmaz Çankaya, Kemalettin Özden

**Affiliations:** 1 Ataturk University, Faculty of Medicine, Department of Infectious Diseases and Clinical Microbiology, Erzurum, Turkey.; 2 Ataturk University, Faculty of Medicine, Department of Radiology, Erzurum, Turkey.

A 58-year-old man presented to the emergency department with severe lumbar pain that had persisted for the previous 10 days. He had a known history of heart valve disease, but no history of surgery. Physical examination revealed restricted vertebral movement and hepatomegaly. 

Laboratory investigations revealed an elevated white cell count (13.45×10^3^/µL), C-reactive protein (CRP) (84.78 mg/L), and erythrocyte sedimentation rate (63 mm/h). Additionally, the patient’s interferon-gamma release test was positive. Based on these findings, empiral ceftriaxone therapy was administered. 

Acid-resistant bacilli were not detected in the patient’s sputum. Rose Bengal, the Wright agglutination test, and Brucella immunoglobulin (Ig)M and IgG tests were negative. 

**FIGURE 1:**
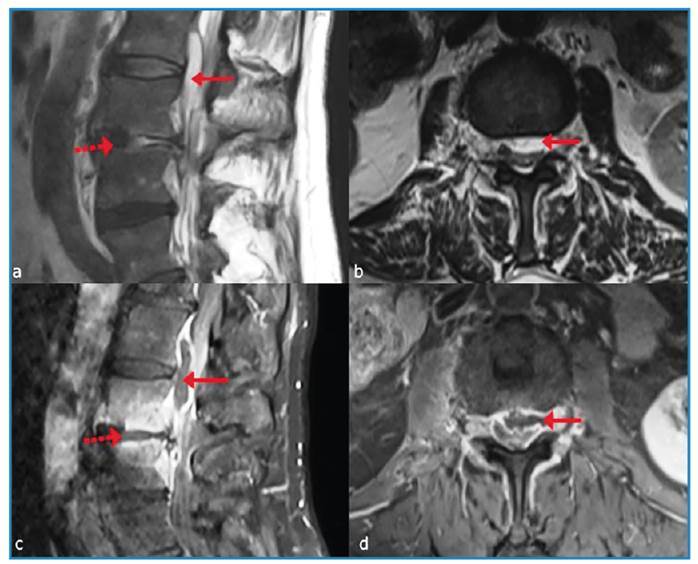
Pre-treatment magnetic resonance imaging showing tuberculous spondylodiscitis and epidural abscess. **(a, b)** Sagittal and axial T2-weighted images with fat suppression (T2 AG) show hyperintense signal intensity in the L3-L4 disc space (dotted arrow), indicating spondylodiscitis. Additionally, an abscess formation is visualized with a long arrow. **(c, d)** Sagittal and axial T1-weighted images with fat suppression and contrast enhancement (T1 AG) demonstrate contrast enhancement in the L3-L4 disc space and vertebral endplates (dotted arrow), suggesting active infection. The peripheral rim of the abscess formation also shows contrast enhancement (long arrow).

The patient’s thoracolumbar magnetic resonance image (MRI) is shown in Figure 1. Unfortunately, the abscess dimensions were unsuitable for drainage. 

After 20 days of antibacterial therapy, no clinical or laboratory improvements were observed. Therefore, antituberculosis therapy with isoniazid 300 mg/day, rifampicin 600 mg/day, ethambutol 2 g/day, and pyrazinamide 2 g/day. 

**FIGURE 2:**
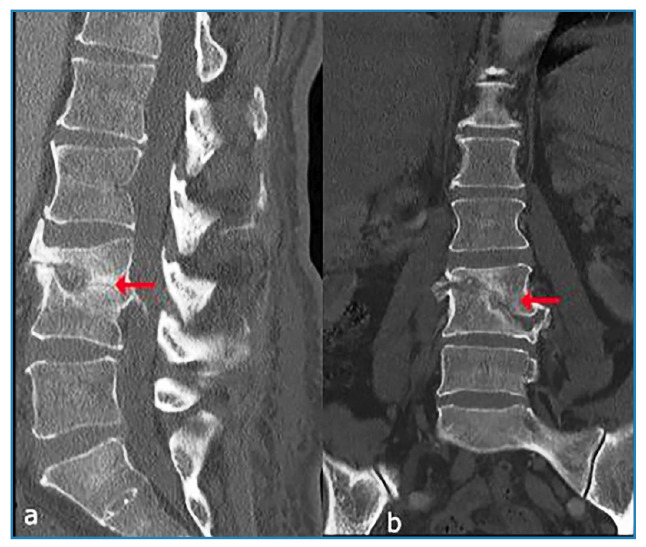
Computed tomography image of post-treatment tuberculous spondylodiscitis sequelae. **(a, b)** Sagittal and coronal images reveal loss of height in the L3 and L4 vertebral bodies, indicating vertebral collapse. Additionally, partial blockage of the vertebral canal is observed (arrow).

During the first month of treatment, CRP decreased to 8.57 mg/L and erythrocyte sedimentation rate to 27 mm/h. The abscess formation contracted significantly by the ninth month of antituberculosis therapy. Due to the insertion of a pacemaker, an MRI could not be performed during this follow-up. However, the patient’s lumbar computed tomography scan is shown in Figure 2. 

Tuberculous spondylodiscitis can be challenging to diagnose due to atypical presentation^1^. Distinguishing it from pyogenic spondylodiscitis is crucial because of their different and specific treatment requirements^2^. 
